# Intrafamilial and extrafamilial sexual assault and its association with alcohol consumption

**DOI:** 10.11606/S1518-8787.2018052000539

**Published:** 2018-11-14

**Authors:** Rubén Valle, Antonio Bernabé-Ortiz, Juan Antonio Gálvez-Buccollini, César Gutiérrez, Silvia S Martins

**Affiliations:** I Universidad de San Martín de Porres. Centro de Investigación en Epidemiología Clínica y Medicina Basada en Evidencias. Lima, Perú; II Instituto Nacional de Salud Mental “Honorio Delgado-Hideyo Noguchi”. DEIDAE de Adultos y Adultos Mayores. Lima, Perú; III Universidad Peruana Cayetano Heredia. CRONICAS Centro de Excelencia de Enfermedades Crónicas. Lima, Perú; IV Universidad Peruana de Ciencias Aplicadas. Facultad de Ciencias de la Salud. Lima, Perú; V Community Psychiatry Associates. California, USA; VI Caremore Care Center. California, USA; VII Universidad de Piura. Facultad de Medicina. Lima, Perú; VIII Columbia University Mailman School of Public Health. Department of Epidemiology. New York, USA

**Keywords:** Adolescent, Exposure to Violence, Child Abuse, Sexual, Domestic Violence, Risk Factors, Socioeconomic Factors, Alcohol Drinking, Health Vulnerability, Adolescente, Exposición a la Violencia, Abuso Sexual Infantil, Violencia Doméstica, Factores de Riesgo, Factores Socioeconómicos, Consumo de Bebidas Alcohólicas, Vulnerabilidad en Salud

## Abstract

**OBJECTIVE:**

Analyze the prevalence of intrafamilial and extrafamilial sexual assault in adolescents of Peru and its association with alcohol consumption.

**METHODS:**

We used a two-step and stratified probabilistic sampling to select male and female students in secondary education from all over Peru. The study included data from 54,756 students with information on demographics, alcohol and drug use, and sexual assault. The statistical analysis considered the complex sampling and we conducted two independent analyses by type of sexual assault (intrafamilial and extrafamilial), stratified by the sex of the victim.

**RESULTS:**

The prevalence of life of intrafamilial sexual assaults (5.4%, 95%CI 5.0–5.8) was similar to that of extrafamilial sexual assaults (6.1%, 95%CI 5.6–6.6). Alcohol consumption in the past year was associated with intrafamilial and extrafamilial sexual assaults that occurred in the same period after adjusting for confounders. Alcohol consumption in the past year was associated with non-physical and physical forms of intrafamilial and extrafamilial sexual assaults in the disaggregated analysis by type of assault. Alcohol consumption in the past year was associated with extrafamilial rape only among females (ORa = 2.8; 95%CI 1.3–5.9).

**CONCLUSIONS:**

Sexual assault against adolescent males and females by family members is a frequent form of victimization that is associated with alcohol consumption in Peru. It is important to examine this form of victimization among adolescents, especially those who consume alcohol.

## RESUMEN

**OBJETIVO:**

Analizar la prevalencia de las agresiones sexuales intrafamiliares y extrafamiliares en adolescentes del Perú y su asociación con el consumo de alcohol.

**MÉTODOS:**

Se utilizó un muestreo probabilístico bietápico y estratificado para seleccionar estudiantes varones y mujeres de educación secundaria de todo el Perú. Los datos de 54,756 estudiantes que incluían información demográfica, sobre consumo de alcohol y drogas, y agresión sexual fueron incluidos en el estudio. En el análisis estadístico se consideró el muestreo complejo y se condujeron dos análisis independientes por el tipo de agresión sexual (intrafamiliar y extrafamiliar), estratificado por el sexo de la víctima.

**RESULTADOS:**

La prevalencia de vida de las agresiones sexuales intrafamiliares (5.4%, IC95% 5.0–5.8) fue similar al de las agresiones extrafamiliares (6.1%, IC95% 5.6–6.6). El consumo de alcohol en el último año se asoció con las agresiones sexuales intrafamiliares y extrafamiliares que ocurrieron en el mismo periodo después de ajustar por los factores de confusión. El consumo de alcohol en el último año se asoció a las formas no físicas y físicas de agresión sexuales intrafamiliares y extrafamiliares en el análisis desagregado por tipo de agresión. El consumo de alcohol en el último año se asoció con las violaciones sexuales extrafamiliares sólo entre las mujeres (ORa = 2.8; IC95% 1.3–5.9).

**CONCLUSIONES:**

La agresión sexual de adolescentes mujeres y varones por miembros familiares es una forma frecuente de victimización que se asocia al consumo de alcohol en el Perú. Es importante indagar por esta forma de victimización en adolescentes, especialmente los que consumen alcohol.

## INTRODUCTION

Sexual assault is any form of sexual contact that occurs without explicit consent of the person and that violates the perception of autonomy of the body. These include a wide range of acts ranging from verbal or visual assaults to sexual assault^1^. The prevalence of life of sexual assaults in adolescents is 6% in Peru^2^. However, some regions have a prevalence of up to 22%^3^. Rape in this country occur mostly in adolescents aged 14–17 years^4^, are preceded by less intrusive assaults^5^, and most occur in the victim’s familial environment (74%)^5^. Therefore, sexual assaults that occur in the familial environment may contribute significantly to the number of cases of sexual victimization among adolescents.

Sexual assaults that occur in the familial environment are called intrafamilial sexual assaults^6^. These assaults are committed by family members or by someone who usually lives in the same home as the victim, such as parents, grandparents, or siblings^6^. Intrafamilial sexual assaults often occur in dysfunctional families in which there is reversal of roles between parents and children and low family cohesion^7^. Although anyone can be sexually assaulted, children are the main victims of this type of assault^6–8^. This may be explained by the fact they spend more time at home and may be more accessible to intrafamilial assaulters^6^, they rely almost completely on an adult who may be their assaulter^9^; and they often do not recognize that they are being assaulted^10^. Intrafamilial sexual assaults have long duration, are associated with major physical and emotional damage to the victim, and generally are not reported^6^ ^,^ ^8^.

On the other hand, sexual assaults committed by people from outside the victim’s familial environment such as friends, lovers or strangers are called extrafamilial sexual assaults^6^. These assaults usually occur outside the household and are mostly committed by people the victim knows and, in a small proportion of cases, by strangers^8^ ^,^ ^11^. Adolescents and young adults are usually victims of this type of assault, mainly because they attend school where they have contact with classmates and teachers and are involved in social activities with greater opportunities of meeting people^6^. In addition, adolescents may participate in activities with sexual risk and consumption of alcohol and drugs, which may make them vulnerable to different forms of violence^12^. Therefore, extrafamilial sexual assaults often become rape or attempted rape and usually occur when the victim has consumed alcohol^13^.

Different studies have found an association between alcohol consumption and sexual assaults^11^ ^,^ ^13^ ^,^ ^14^. These works implicitly study extrafamilial sexual assaults, since they evaluate assaults committed by strangers, friends, or lovers that occur in schools, universities, or parties^11^ ^,^ ^15^ ^,^ ^16^. These studies work with the assumption that a person increases their vulnerability to assault if they consume alcohol and if there is a perpetrator around them^17^. Alcohol seems to only increase the risk, while the presence of the assaulter is the determining factor so the assault is carried out^18^. These two factors also act in intrafamilial assaults but in different ways. In this case, the perpetrator has total proximity in time and space with the victim and, hence, needs no substance that increases the vulnerability of this victim. Therefore, alcohol consumption by the victim does not appear to precede the sexual assault, but it seems the victim consumes this substance as a measure to relieve the stress caused by the assault^19^. Thus, the direction of the association is different depending on the type of assault.

Investigations into intrafamilial sexual assaults have focused mainly on samples of children^7^ ^,^ ^9^. Studies that evaluate this form of assault in adolescents are scarce, although this is one of the main forms of sexual victimization in Peru^5^. In addition, while the relationship between alcohol consumption and sexual assault in general has been studied, the association between alcohol consumption and intrafamilial and extrafamilial sexual assault has not been studied specifically.

The objective of this study was to analyze the prevalence of intrafamilial and extrafamilial sexual assault in Peruvian adolescents and its association with alcohol consumption.

## METHODS

Analysis of the database of the *Tercer Estudio Nacional: Prevención y consumo de drogas en estudiantes de secundaria 2009* (Third National Study: Prevention and consumption of drugs in high school students 2009)^2^. This study surveyed high school students residing in urban areas in all Peru (cities with over 30,000 inhabitants) to estimate the prevalence and characteristics of the consumption of alcohol and drugs. The two-step sampling was probabilistic, stratified and independent of each domain of study. The stratum was type of school (public or private) and the primary and secondary sampling units were schools and classes, respectively. Data was collected with anonymous and self-administered questionnaire in the classroom during regular school periods in the absence of school staff. Further details on the sampling and the process of selection of participants can be found in the report of the study^2^.

The variables intrafamilial and extrafamilial sexual assault were evaluated using the following questions: “Have you ever in your life been sexually assaulted within your family?”; and “Outside your family, have you ever in your life been sexually assaulted?”. Immediately after these questions, we provided examples of sexual assault in order to clarify the concept for participants: Let’s say, has anyone touched you, wanted to touch your private parts, wanted to kiss you, etc. Participants who responded affirmatively, subsequently answered questions about the occurrence of such incidents in the past year, the identity of the assaulters, and the form of victimization. The alcohol consumption variable was evaluated using the question “Have you ever in your life consumed alcohol?”. Having responded affirmatively, participants answered questions about alcohol consumption in the past year and about the frequency of consumption. The frequency of consumption was classified into: no alcohol consumption, once a year, and two or more times a year.

The study considered other variables of the individual, relational, and social sphere of the individual that could influence the association of study, according to the ecological model of violence of the World Health Organization^20^. These included age, school, grade, appropriate degree for the age, use of illicit drugs, type of school by sex, family structure, and presence of family member with criminal history. Use of illicit drugs was evaluated using the question: “Have you ever consumed any illicit drugs?”. The options available were marijuana, base paste of cocaine, cocaine, ecstasy, meth, inhalants, hallucinogens, and substances recognized by respondents as illicit. This question was reduced to a binary variable (No or Yes). The appropriate grade for the age variable was created considering the participants’ current age and expected age in relation to the current study grade (first grade = 12, second grade = 13, third grade = 14, fourth grade = 15, and fifth grade = 16). This variable was categorized as “appropriate,” unless the current age was two or more years above the expected age for the study grade.

The analysis used Taylor series estimation methods considering the type of complex sampling of the original study. We conducted two separate analyses by type of sexual assault: intrafamilial and extrafamilial, stratified by sex of victim, given the differences found between females and males in the association between alcohol consumption and sexual assault^21^. Extrafamilial sexual assault in the past year was analyzed as dependent variable in relation to alcohol consumption in the past year (independent variable), and intrafamilial sexual assault in the past year was analyzed as independent variable in relation to alcohol consumption (dependent variable). The association was estimated using logistic regression models to estimate odds ratios adjusted (ORa) and 95% confidence interval (95%CI) where all covariates were included in the final model. We selected the variables entered in the final model based on a causal acyclic diagram. The sexual assault variable was disaggregated into three variables considering the degree of intrusion of the assault: non-physical sexual assault (they have shown you the genitals and you have been watched while bathing), physical sexual assault (inappropriate touching, they have kissed you by force, and they have kissed you or touched your breasts, genitals, or buttocks), and rape (they have forced you to have sex and they have drugged you to force you to have sex). We conducted a sub-analysis between alcohol consumption and the three disaggregated variables of sexual assault to estimate more specific associations. Statistical analysis used the program Stata v.12 (*Stata Corporation, College Station*, Texas, EE.UU.).

This study was approved by the Institutional Committee of Ethics of the Universidad Peruana Cayetano Heredia.

## RESULTS

Participation rate was 81.3%. Data from 54,756 participants aged 11‒17 years were included in the analysis (50.2% female). Mean age was 14.4 years [standard deviation (SD) = ±1.5 years]. The participants’ sociodemographic characteristics by type of assault and sex of victim are presented in Table 1.

**Table 1:** Prevalence of life of sexual assaults in adolescents according to sociodemographic characteristics. Peru, 2009.

Variable	Intrafamilial		Extrafamilial	
	
	Female (%)	Male (%)	Female (%)	Male (%)
Age (years)				
11–13	4.6	4.8	5.5	6.5
14–17	6.6	4.8	6.7	5.6
School				
Private	5.8	3.8	5.0	3.6
Public	5.9	5.2	6.7	6.8
Grade				
First	4.9	5.5	6.2	8.2
Second	5.0	4.6	6.2	5.7
Third	6.5	4.5	6.2	5.1
Fourth	6.3	5.3	6.4	5.6
Fifth	7.6	3.9	6.6	3.9
Age-appropriate grade				
Appropriate	5.8	4.6	6.0	5.5
Older	7.8	7.1	9.2	9.6
Use of illicit drugs				
No	5.1	3.2	5.4	4.3
Yes	19.7	19.5	21.3	19.9
Type of school by sex				
Female	5.7	NA	5.0	NA
Male	NA	4.9	NA	6.4
Mixed	6.0	4.8	6.5	5.8
Family composition				
Both parents	5.2	4.4	5.5	5.5
Father	6.7	6.8	9.0	5.9
Mother	5.9	4.6	6.7	5.9
Father and partner	8.9	6.6	4.4	8.1
Mother and partner	10.8	4.7	10.1	7.7
No parent	7.9	7.5	8.5	7.5
Partner and with parents or in-laws	16.6	10.1	20.2	9.3
Partner	18.3	14.9	22.3	15.0
Relative with criminal record				
No	5.0	4.1	5.1	5.1
Yes	8.5	6.7	9.5	7.9

NA: It does not apply

Percentages (%) were calculated based on the row totals.

%: Weighted percentage

The prevalence of life of sexual assaults (global) was 9.1% (95%CI 8.5–9.6) with prevalence in females of 9.8% (95%CI 9.3–10.5) and in males of 8.2% (95%CI 7.5–8.9). The prevalence of life of intrafamilial sexual assaults was 5.4% (95%CI 5.0–5.8), with prevalence in females of 5.9% (95%CI 5.5–6.4) and in males of 4.8% (95%CI 4.3–5.4). The prevalence of life of extrafamilial sexual assaults was 6.1% (95%CI 5.6–6.6), with prevalence in females of 6.3% (95%CI 5.8–6.8) and in males of 5.9% (95%CI 5.3–6.6). The prevalence of sexual assaults in the past year was 3.3% (95%CI 2.9–3.6), with prevalence in females of 3.2% (95%CI 2.9–3.6) and in males of 3.3% (95%CI 2.9–3.8). The prevalence of intrafamilial sexual assaults in the past year was 1.9% (95%CI 1.7–2.2), with prevalence in females of 1.8% (95%CI 1.5–2.1) and in males of 2.1% (95%CI 1.7–2.4). The prevalence of extrafamilial sexual assaults in the past year was 2.3% (95%CI 2.0–2.6), with prevalence in females of 2.1% (95%CI 1.9–2.4) and in males of 2.4% (95%CI 2.0–2.8).

The victims experienced a wide range of assaults, from non-physical sexual assaults to rape. Inappropriate touching was the assault most reported by females and males in intrafamilial sexual assaults. A higher percentage of males (24.9%) than females (18.9%) reported that the assaults occurred frequently in the past year. Other forms of sexual assault that were not included in the survey were the most frequent in relation to extrafamilial sexual assaults in both sexes, followed by forced kissing in the case of females and being shown the genitals in the case of males. The frequency of these incidents was designated as “frequent” by 19.2% of females and 32.9% of males (Table 2).

**Table 2:** Characteristics of the sexual assaults. Peru, 2009.

Variable	Intrafamilial		Extrafamilial	
	
	Female (%)	Male (%)	Female (%)	Male (%)
Type of sexual assaults*				
They have shown you the genitals	9.1	9.2	10.3	12.7
You have been watched while bathing	8.0	11.1	8.7	10.7
You have been subject to touching on your body	20.8	13.9	15.9	9.4
They have kissed you or wanted to kiss you by force	11.3	8.9	17.1	9.7
They have kissed you or touched your breasts or genitals	7.4	5.0	5.9	6.6
They have forced you to have sex	6.3	4.8	6.7	3.9
You have been drugged for having sex	1.7	2.9	1.5	4.1
Other	9.6	12.1	39.4	47.2
No answer	33.4	32.9	0	0
Frequency (in the last year)				
Some times	81.1	75.1	80.8	67.2
Frequently	18.9	24.9	19.2	32.9
Number of different assaulters				
One	72.6	76.9	76.7	75.5
More than one	2.9	1.1	1.9	0.9
No answer	24.5	22.0	21.4	23.6
Intrafamilial assaulters				
Parents	16.9	25.0	NA	NA
Stepparents	11.6	18.4	NA	NA
Sibling	6.5	9.9	NA	NA
Grandparents	4.0	4.3	NA	NA
Uncle/aunt	16.9	2.8	NA	NA
Other	22.5	18.8	NA	NA
No answer	24.5	22.0	NA	NA
Extrafamilial assaulters				
Neighbor	NA	NA	10.3	10.8
Schoolmate/friend	NA	NA	24.9	30.2
Internet friend	NA	NA	3.6	8.5
Lover	NA	NA	8.6	8.7
Teacher	NA	NA	1.6	2.9
Stranger	NA	NA	15.0	3.9
Other	NA	NA	16.9	12.9
No answer	NA	NA	21.4	23.6

NA: It does not apply

*Multiple answer.

%: Weighted percentages

The prevalence of alcohol consumption in the past year was 24.4% (95%CI 23.2–25.7), with prevalence in females of 22.2% (95%CI 20.7–23.8) and in males of 26.7% (95%CI 25.4–28.1). A total of 52.9% (95%CI 48.8–56.9) of the victims of intrafamilial sexual assaults and 49.0% (95%CI 45.1–52.9) of the victims of extrafamilial sexual assaults occurred in the past year reported having consumed alcohol in the same period (Table 3).

**Table 3:** Frequency of sexual assaults among the participants who reported having consumed alcohol in the past year. Peru, 2009.

Consumption of alcohol in the past year	Intrafamilial				Extrafamilial			
	
	Female		Male		Female		Male	
			
	%	95%CI	%	95%CI	%	95%CI	%	95%CI
Alcohol consumption								
No	1.2	1.0–1.4	1.2	1.0–1.5	1.5	1.3–1.7	1.6	1.3–1.9
Yes	3.9	3.2–4.8	4.4	3.6–5.3	4.5	3.8–5.3	4.5	3.7–5.5
Frequency of consumption								
No	1.2	1.0–1.4	1.2	1.0–1.5	1.5	1.3–1.7	1.6	1.3–1.9
Once a year	3.4	2.6–4.5	4.2	3.2–5.5	4.1	3.2–5.2	4.2	3.3–5.3
≥ 2 times a year	4.3	3.3–5.7	4.5	3.6–5.7	4.8	3.9–5.9	4.7	3.6–6.1

Percentages (%) were calculated based on the row totals.

%: Weighted percentages

In general, intrafamilial sexual assaults occurred in the past year were associated with alcohol consumption in the past year after adjusting for confounders. Moreover, alcohol consumption in the past year was associated with extrafamilial sexual assaults in the same period after adjusting for confounders. Disaggregated analysis showed that non-physical and physical intrafamilial sexual assaults were associated with alcohol consumption in students of both sexes; however, intrafamilial rape was not associated with alcohol consumption. On the other hand, alcohol consumption in the past year was associated with non-physical and physical extrafamilial sexual assaults in students of both sexes. However, we observed that alcohol consumption in the past year was associated with extrafamilial rape only among females (ORa = 2.8; 95%CI 1.3–5.9). The association between alcohol consumption and sexual assaults did not differ by the frequency of alcohol consumption (Tables 4 and 5).

**Table 4:** Associationa between alcohol consumption in the past year and intrafamilial sexual assaultsb that occurred in the past year (global and disaggregated measurements). Peru, 2009

Sexual assaults	Consumption of alcohol in the past year				Frequency of consumption				Frequency of consumption			
	
					(once a year)				(≥ 2 times a year)			
		
	Female		Male		Female		Male		Female		Male	
					
	ORa	95%CI	ORa	95%CI	ORa	95%CI	ORa	95%CI	ORa	95%CI	ORa	95%CI
Sexual assault (global)												
No	1	Ref	1	Ref	1	Ref	1	Ref	1	Ref	1	Ref
Yes	2.3	1.7–3.2	2.4	1.9–3.2	2.4	1.7–3.3	2.7	1.9–3.7	2.3	1.5–3.5	2.3	1.7–3.2
Non-physical												
No	1	Ref	1	Ref	1	Ref	1	Ref	1	Ref	1	Ref
Yes	2.6	1.6–4.4	2.9	1.9–4.6	3.1	1.7–5.6	3.2	1.9–5.4	2.1	1.1–4.2	2.7	1.7–4.5
Physical												
No	1	Ref	1	Ref	1	Ref	1	Ref	1	Ref	1	Ref
Yes	2.1	1.3–3.3	2.8	1.9–4.2	1.8	1.1–3.2	2.5	1.3–4.6	2.1	1.1–3.8	3.1	1.9–4.9
Rape												
No	1	Ref	1	Ref	1	Ref	1	Ref	1	Ref	1	Ref
Yes	1.6	0.6–4.3	1.8	0.5–5.7	0.7	0.2–2.2	1.7	0.3–10.2	2.3	0.7–7.5	1.4	0.4–4.5

Ref: Reference; ORa: Odds ratio adjusted (final model)

^a^ The model was adjusted for age, school, grade, appropriate grade for the age, use of illicit drugs, type of school according to sex, family composition, and family member with criminal history.

^b^ Non-physical sexual assault: they have shown you the genitals and you have been watched while bathing; Physical sexual assault: you have been touched on your body, they have kissed you or wanted to kiss you by force, and they have kissed or touched your breasts or genitals; Rape: they have forced you to have sex and you have been drugged for having sex.

**Table 5:** Associationa between alcohol consumption in the past year and extrafamilial sexual assaultsb in the past year (global and disaggregated measurements). Peru, 2009.

Sexual assaults	Consumption of alcohol in the past year				Frequency of consumption				Frequency of consumption			
	
					(Once a year)				(≥ 2 times a year)			
		
	Female		Male		Female		Male		Female		Male	
					
	ORa	95%CI	ORa	95%CI	ORa	95%CI	ORa	95%CI	ORa	95%CI	ORa	95%CI
Sexual assault (global)												
No	1	Ref	1	Ref	1	Ref	1	Ref	1	Ref	1	Ref
Yes	2.3	1.8–3.1	2.3	1.7–2.9	2.4	1.7–3.3	2.2	1.7–3.0	2.3	1.7–3.1	2.3	1.6–3.2
Non-physical												
No	1	Ref	1	Ref	1	Ref	1	Ref	1	Ref	1	Ref
Yes	2.1	1.3–3.4	2.0	1.4–2.9	2.1	1.2–3.8	2.3	1.4–3.6	2.1	1.2–3.7	1.7	1.1–2.5
Physical												
No	1	Ref	1	Ref	1	Ref	1	Ref	1	Ref	1	Ref
Yes	2.4	1.6–3.5	3.3	2.2–4.9	2.3	1.5–3.6	2.9	1.7–4.9	2.4	1.5–3.8	3.6	2.2–5.9
Rape												
No	1	Ref	1	Ref	1	Ref	1	Ref	1	Ref	1	Ref
Yes	2.8	1.3–5.9	1.2	0.5–2.6	3.0	1.2–7.7	0.3	0.1–1.1	2.6	1.1–6.8	1.8	0.7–4.2

Ref: Reference; ORa: Odds ratio adjusted (final model)

^a^ The model was adjusted for age, school, grade, appropriate grade for the age, use of illicit drugs, type of school according to sex, family composition, and family member with criminal history.

^b^ Non-physical sexual assault: they have shown you the genitals and you have been watched while bathing; Physical sexual assault: you have been touched on your body, they have kissed you or wanted to kiss you by force, and they have kissed or touched your breasts or genitals; Rape: they have forced you to have sex and you have been drugged for having sex

## DISCUSSION

The prevalence of life of intrafamilial sexual assaults was similar to that of extrafamilial sexual assaults and alcohol consumption in the past year in females and males was associated with intrafamilial sexual assaults in the same period. Alcohol consumption of males and females was associated with non-physical and physical forms of intrafamilial and extrafamilial sexual assaults occurred in the past year. However, alcohol consumption was associated with extrafamilial rape only among females.

The similar prevalence of life between intrafamilial and extrafamilial sexual assaults found in our study differs from studies that show a greater frequency of extrafamilial sexual assaults than intrafamilial sexual assaults in underage individuals^6^ ^,^ ^8^ ^,^ ^22^. In Peruvian adolescents, some family environments can also constitute a risk factor for the occurrence of sexual assaults; which partially explains why most rape in Peru are intrafamilial^5^. Moreover, given that intrafamilial sexual assaults are characterized by early start in the life of the person and occur repeatedly^6^ ^,^ ^8^, it is likely that the reported incidents began early in childhood and persisted through adolescence. On the other hand, given that this type of assault is not reported for the most part, it is unlikely that these cases have received proper medical and legal handling^8^ ^,^ ^22^. Intrafamilial sexual assaults deserve special attention in the context under study not only for their magnitude, but also for the consequences associated with their occurrence.

Although some studies find no difference in the frequency of sexual assaults in relation to sex of victim^23^ ^,^ ^24^, our results are consistent with studies that show higher prevalence of sexual assaults in females than in males^21^ ^,^ ^25^ ^,^ ^26^. However, in evaluating the prevalence by type of assault, it is found that intrafamilial sexual assaults were more frequent in females than in males, but just as prevalent in the case of extrafamilial assaults. These results are consistent with the literature that shows that sexual assaults committed by family members are more frequent in females than in males, but differ from the results that show that extrafamilial sexual assaults are more frequent in females^6^ ^,^ ^8^. The differences found, although significant, are small, so they may not be important from a public health perspective. In addition, since males tend to not report the assaults to a greater extent than females^27^, there is likely greater underestimation of the prevalence of sexual assaults in males.

The association between alcohol consumption and extrafamilial sexual assaults has been shown in previous studies, but not the association with intrafamilial sexual assaults. In extrafamilial sexual assaults, alcohol precedes sexual assault and increases the vulnerability of the person due to the effects at cognitive and motor level it produces^28^ ^,^ ^29^. On the other hand, based on the concept that victims of sexual assaults tend to consume alcohol as a form of self-medication^19^, intrafamilial sexual assault was considered as an independent variable of alcohol consumption. This analysis showed an association between both variables and was based on a conceptual criterion for the characteristics of intrafamilial sexual assaults. However, given the cross-sectional design of the study, this result may be caused by a third factor related to both variables (example: family dysfunction)^18^. Future studies should evaluate the role of alcohol in intrafamilial sexual assaults.

Alcohol was associated with intrafamilial and extrafamilial sexual assaults of the non-physical and physical types in the disaggregated analysis. However, alcohol consumption was associated with extrafamilial rape only in females. On the other hand, intrafamilial rape in females was not associated with alcohol consumption. That is, female victims of these assaults did not report having consumed alcohol more frequently than females who were not sexually assaulted. In addition, alcohol consumption by males was not associated with intrafamilial or extrafamilial rape. This result suggests that rape is independent of alcohol consumption in males. Given that less intrusive forms of intrafamilial and extrafamilial sexual assaults were associated with alcohol, it is noteworthy that rape is not associated. The lack of association found in this sub-analysis may be caused by a low statistical power as a result of the different stratifications of the sample.

The study shows limitations. Alcohol consumption and sexual assaults are susceptible to the social desirability bias, because the report of both events may be underestimated^30^. In addition, a causal relationship cannot be established between alcohol consumption and sexual assaults, in any direction, due to the cross-sectional design of the study. A high percentage (21–25%) of victims of intrafamilial sexual assaults did not report information on the type of assault, possibly for considering the legal implications that could be brought by the disclosure of such information. On the other hand, the study did not collect information on alcohol consumption at the time of the sexual assault, which would have enabled to determine the role of alcohol in intrafamilial and extrafamilial assaults. Since we only evaluated high school students of urban areas, the findings cannot be generalized to adolescents of rural areas or in school age who do not attend school, for which the frequency of alcohol consumption and sexual assaults may be different.

Despite these limitations, the study has strengths: it uses data from a population-based study conducted throughout Peru and analyzes sexual assaults on males, a group for which there is little information in Latin America. In conclusion, sexual assaults committed by family members are frequent forms of sexual victimization in female and male adolescents of Peru. The occurrence of these incidents is associated with alcohol consumption, as has been observed with extrafamilial sexual assaults. Therefore, it is suggested the creation of appropriate channels to facilitate the notification of intrafamilial and extrafamilial sexual assault for children and adolescents. Programs for prevention of sexual violence and alcohol consumption in adolescents should actively investigate the occurrence of these incidents by the co-occurrence that may exist between both events. Future studies should evaluate the factors that determine the occurrence of intrafamilial sexual assaults in adolescents.
